# In Vitro and In Vivo Antibacterial Efficacy of a Ciprofloxacin Delivery System Based on *Streptococcus suis* Extracellular Vesicles

**DOI:** 10.3390/ani16142262

**Published:** 2026-07-22

**Authors:** Wenjie Jin, Zhiheng Chang, Yahao Yu, Aoqi Zhan, Shenao Song, Yuxin Wang, Baobao Liu, Yang Wang, Li Yi

**Affiliations:** 1 College of Life Science, Luoyang Normal University, Luoyang 471934, China; 2 College of Animal Science and Technology, Henan University of Science and Technology, Luoyang 471000, China; 3 Henan Provincial Engineering Research Center for Detection and Prevention and Control of Emerging Infectious Diseases in Livestock and Poultry, Luoyang 471003, China

**Keywords:** extracellular vesicles, antibiotic delivery, intracellular bacteria, efflux pump, *Streptococcus suis*, *Salmonella enterica* serovar Typhimurium

## Abstract

Conventional antibiotics exhibit limited ability to penetrate host cell membranes, making intracellular bacterial infections difficult to eradicate completely. As naturally derived nanoscale membrane structures, bacterial extracellular vesicles (EVs) possess excellent biocompatibility and intrinsic transmembrane transport capability, thereby demonstrating unique advantages for in vivo drug delivery. The present study investigated the feasibility of using EVs derived from the avirulent *Streptococcus suis* T15 as novel carriers for ciprofloxacin delivery. We also comprehensively evaluated the biosafety and anti-infective efficacy of this nanodrug delivery system in vitro and in vivo. Cytotoxicity assays, live/dead cell staining, and hemolysis analyses demonstrated that T15-derived EVs at concentrations below 50 μg/mL did not cause significant cellular damage or hemolysis. Serum biochemical analyses in mice further confirmed the absence of obvious organ toxicity, indicating favorable biosafety within the tested concentration range. Ciprofloxacin was successfully loaded into EVs using a combination of ultrasonication and electroporation, achieving a drug concentration of 438.6 μg/mL and a loading efficiency of 10.96%. The ciprofloxacin-loaded EVs (EV-CIP) exhibited significantly greater antibacterial activity than free ciprofloxacin against both intracellular bacteria and fluoroquinolone-resistant strains exhibiting efflux pump activity. Evaluation in animal infection models showed that EV-CIP markedly reduced mortality in infected *Galleria mellonella* larvae. It also decreased bacterial burdens in multiple mouse organs and significantly alleviated histopathological damage. These results collectively suggest that EVs derived from the avirulent *S. suis* T15 were safe and effective within the tested concentration range and experimental conditions. The EV-based ciprofloxacin delivery system substantially enhanced the clearance of intracellular pathogens and fluoroquinolone efflux pump-positive bacteria, suggesting its potential application in the treatment of difficult-to-treat bacterial infections. This study provides a theoretical and experimental basis for the further development of novel EV-based anti-infective drug delivery strategies for livestock and poultry.

## Simple Summary

Bacterial infections remain a major challenge to animal health and productivity in livestock production systems and cause substantial economic losses. Certain pathogenic bacteria can survive and proliferate within host cells. Conventional antibiotics often fail to reach effective intracellular concentrations, which may contribute to persistent infections and an increased risk of antimicrobial resistance. In the present study, we developed a ciprofloxacin delivery system using extracellular vesicles (EVs) derived from the avirulent *Streptococcus suis* T15. We then systematically evaluated its biosafety and antibacterial efficacy. The results demonstrated that T15-derived EVs had a favorable safety profile within the tested concentration range. Furthermore, compared with free ciprofloxacin, EV-delivered ciprofloxacin displayed significantly enhanced antibacterial activity against both intracellular bacteria and efflux pump-mediated fluoroquinolone-resistant strains. In animal infection models, the EV-based delivery system effectively reduced bacterial burdens, alleviated tissue damage, and improved host survival rates. Taken together, these results support the potential of T15-derived EVs as an antibiotic delivery platform. This system may provide a promising strategy for treating intracellular bacterial infections and enhancing the activity of ciprofloxacin against efflux pump-positive strains.

## 1. Introduction

Bacterial infections remain a major threat to animal health and productivity in intensive livestock production systems. Numerous intracellular pathogens are capable of surviving and replicating within host cells, where the host cell membrane serves as a natural protective barrier that restricts the intracellular penetration of antibiotics. As a result, substantial concentration gradients arise between the extracellular and intracellular compartments, preventing intracellular antibiotics from reaching effective bactericidal levels and markedly compromising their antibacterial efficacy. Consequently, intracellular pathogens are often difficult to eradicate in clinical settings [1,2]. A variety of bacterial pathogens can survive within macrophages, epithelial cells, or endothelial cells, thereby establishing relatively concealed intracellular reservoirs that enable evasion of host immune clearance [3,4]. At present, antibiotics remain the primary therapeutic strategy for the treatment of bacterial infections. However, insufficient antimicrobial concentrations within the infectious microenvironment, the emergence of antibiotic resistant strains, and the intracellular persistence of pathogens frequently contribute to therapeutic failure and recurrent infection [5]. Previous studies have demonstrated that the intracellular antibacterial activity of antibiotics is determined not only by their minimum inhibitory concentrations measured in vitro, but also by their capacity for cellular uptake, subcellular localization, intracellular retention, and release kinetics [6]. Collectively, these challenges highlight the urgent need to develop novel strategies for the intracellular delivery of antibiotics.

Current strategies to enhance the intracellular delivery of antibiotics mainly fall into two categories. The first involves the use of adjuvants to increase cell membrane permeability; however, this approach is often limited by variable and unpredictable efficacy [7,8]. The second strategy relies on engineered delivery vehicles that interact with the host cell surface to bypass cellular barriers and transport high local concentrations of antibiotics into intracellular compartments [9]. Common carrier based delivery systems include liposomes, nanoparticles and polymeric hydrogels [10]. Among these delivery systems, liposomes have become a major focus of drug delivery research owing to their excellent biocompatibility, tunable properties, and sustained drug release capability [11]. However, they still exhibit several limitations, including poor structural stability and susceptibility to premature drug leakage [12,13].

Bacteria-derived extracellular vesicles (BEVs), nanoscale structures enclosed by lipid bilayers and secreted by bacteria, have emerged as natural nanocarrier systems [14]. BEVs are capable of protecting their cargo from environmental degradation and exhibit greater stability than many synthetic lipid-based delivery systems [15]. Previous studies have demonstrated that BEVs can interact with host cells or bacteria through multiple mechanisms, including endocytosis and membrane fusion, thereby facilitating the intracellular delivery of their cargo into host cells [16]. In addition, EVs possess favorable biocompatibility, and bacterial outer membrane vesicles have increasingly been investigated as vaccine platforms, immunomodulatory agents, and drug delivery vehicles in recent years [17,18]. Notably, recent evidence suggests that bacterial uptake of extracellular vesicles is selective, with bacteria exhibiting a preference for vesicles derived from the same or closely related species. This property confers an intrinsic targeting capability on BEVs, further highlighting their potential as precision delivery platforms [14,19].

Based on these considerations, we sought to investigate the potential of extracellular vesicles derived from the avirulent *Streptococcus suis* T15 as a natural delivery platform for ciprofloxacin. To this end, we developed a ciprofloxacin-loaded EV delivery system and systematically evaluated its antibacterial efficacy against intracellular pathogens as well as fluoroquinolone efflux pump-positive strains. The aim of this study was to evaluate whether EV-mediated delivery could enhance the antibacterial efficacy of ciprofloxacin against intracellular pathogens and fluoroquinolone efflux pump-positive bacterial strains.

## 2. Materials and Methods

### 2.1. Bacterial Strains and Culture Conditions

The *Streptococcus suis* (*S. suis*) T15 was used for extracellular vesicle isolation, whereas *S. suis* HA9801, *S. suis* QD, and *Salmonella enterica* serovar Typhimurium SL1344 were used for in vitro and in vivo bactericidal assays. *S. suis* strains were cultured in tryptic soy broth (TSB), while *Salmonella enterica* serovar Typhimurium SL1344 was cultured in Luria–Bertani (LB) medium. All bacterial strains were incubated at 37 °C with shaking at 180 rpm.

### 2.2. Isolation and Purification of Extracellular Vesicles

Bacterial extracellular vesicles were isolated from culture supernatants using a combination of ultracentrifugation and size exclusion chromatography (SEC), according to the method described by Li et al. with slight modifications [20]. Briefly, bacteria were cultured at 37 °C with shaking at 180 rpm for 24 h, and the resulting bacterial cultures were used for EV extraction. The cultures were centrifuged at 10,000 *g* for 20 min at 4 °C to remove bacterial cells. The collected supernatants were subsequently filtered through 0.45 μm and 0.22 μm membrane filters. The filtrates were then concentrated using a tangential flow ultrafiltration system, followed by ultracentrifugation at 100,000 *g* for 2 h at 4 °C. After centrifugation, the supernatants were discarded, and the resulting pellets were resuspended in sterile PBS, yielding crude EV preparations. The crude EV suspensions were slowly loaded onto pre-equilibrated SEC columns, after which sterile PBS was added for elution. Fractions corresponding to 4–7 mL of eluate were collected and further concentrated using 100 kDa ultrafiltration tubes. Purified EV samples were stored at 4 °C for short-term use and at −80 °C for long-term storage.

### 2.3. Characterization of Extracellular Vesicles

The size distribution and particle concentration of the isolated EVs were determined using a ZetaView nanoparticle tracking analyzer (ZetaView PMX 110, Particle Metrix, Inning am Ammersee, Germany), according to the method described by Li et al. with slight modifications [21]. Prior to analysis, EV samples were diluted to an appropriate concentration with sterile PBS, and the PBS used as the diluent was measured separately to exclude potential contamination. The diluted EV suspensions were then loaded into the sample chamber for nanoparticle tracking analysis.

### 2.4. Cell Culture

Three cell lines were used in this study: the human laryngeal epithelial carcinoma cell line Hep-2, the murine macrophage-like leukemia cell line Raw264.7, and the human non-small cell lung carcinoma cell line A549. All cell lines were cultured in complete DMEM supplemented with 10% heat-inactivated FBS. Cells were maintained in a humidified incubator at 37 °C with 5% CO_2_.

### 2.5. Microscopic Observation of Extracellular Vesicle Internalization

Microscopic observation of EV internalization was performed according to the method described by Mehanny et al. with slight modifications [22]. Briefly, cells were seeded into 96-well plates at a density of 5 × 10^3^ cells per well and cultured at 37 °C in a humidified atmosphere containing 5% CO_2_ until a confluent monolayer was formed. EVs were labeled with FM4-64 at a final concentration of 1 μM by incubation at 37 °C for 20 min in the dark. To remove unbound dye, the labeled EV suspensions were centrifuged using 100 kDa ultrafiltration tubes at 3000 *g* for 10 min, followed by the addition of PBS and repeated centrifugation for a total of three washing cycles. The labeled EVs at different concentrations were then incubated with cultured Raw264.7 cells for 4 h at 37 °C in a 5% CO_2_ incubator. To remove EVs adhering to the cell surface, cells were treated for 45 s with a solution containing 0.5 mM sodium chloride and 0.5% ice-cold acetic acid, followed by three washes with PBS. Subsequently, cells were stained with FM1-43 at a final concentration of 1 μM for 15 min in the dark, after which the dye was removed, and the cells were washed three times with PBS. The washed cells were fixed with paraformaldehyde for 15 min at room temperature and rinsed three times with PBS. Cell nuclei were then stained with 100 μg/mL DAPI for 20 min, followed by three additional washes with PBS. Finally, the 96-well plates were mounted on the microscope stage and observed using an inverted fluorescence microscope.

### 2.6. Evaluation of Extracellular Vesicle Cytotoxicity

The cytotoxicity of EVs was evaluated using a previously established laboratory protocol with slight modifications [23]. Briefly, cells were cultured in 96-well plates at 37 °C in a humidified incubator containing 5% CO_2_ until a confluent monolayer was formed. The culture medium was then removed, and the cells were washed with PBS before the addition of maintenance medium consisting of DMEM supplemented with 2% FBS. EVs at the indicated concentrations were subsequently added to the cells and incubated for 12 h under standard culture conditions. Cells treated with an equal volume of PBS, representing 100% viability, were used as the negative control. Following incubation, 10 μL of CCK-8 solution was added to each well, and the plates were further incubated for 1 h in the dark. Absorbance was then measured at 450 nm using a microplate reader.

### 2.7. Calcein-AM/PI Dual Staining Assay for Live and Dead Cells

The assay was performed according to the method described by Wu et al. [24]. Briefly, cells were cultured in DMEM supplemented with 10% fetal bovine serum (FBS) until a confluent monolayer was formed. The culture medium was then removed, and the cells were washed with PBS. Subsequently, maintenance medium consisting of DMEM supplemented with 2% FBS was added, and the cells were incubated with different concentrations of EVs for 24 h at 37 °C in a humidified atmosphere containing 5% CO2. Following treatment, cells were stained with Calcein-AM and propidium iodide (PI) for 30 min according to the manufacturer’s instructions. Excess staining solution was discarded, and the cells were washed three times with PBS before observation under a fluorescence microscope.

### 2.8. Hemolysis Assay

The hemolytic activity assay was performed according to the method described by Wang et al. with slight modifications [25]. Fresh rabbit whole blood was centrifuged at 3000 *g* for 10 min, after which the supernatant was carefully discarded. The erythrocyte pellet was resuspended in an equal volume of sterile PBS and centrifuged again. This washing procedure was repeated twice to obtain purified erythrocytes. The resulting erythrocytes were diluted with sterile PBS to prepare a 5% erythrocyte suspension and aliquoted into sterile centrifuge tubes. The 5% erythrocyte suspension was incubated with EVs at a final concentration of 50 μg/mL at 37 °C for 1 h. A 1% Triton X-100 solution was included as a positive control to induce complete hemolysis. Following incubation, the samples were centrifuged at 3000 *g* for 10 min, and 200 μL of the supernatant was transferred into a 96-well plate. Absorbance at 570 nm was measured using a full-wavelength microplate reader (Infinite 200 PRO, Tecan Austria GmbH, Grödig, Austria).

### 2.9. Analysis of Serum Biochemical Parameters in Mice

Serum biochemical analysis was performed according to the method described by Qu et al. with slight modifications [26]. Ten mice were randomly divided into two groups. The control group received an injection of 200 μL PBS, whereas the experimental group received 200 μL EVs at a concentration of 50 μg/mL. After 24 h, blood samples were collected from the retro-orbital venous plexus. The samples were allowed to stand at 37 °C for 2 h and were subsequently centrifuged at 3000 rpm for 10 min to obtain serum. The collected serum samples were processed according to the manufacturer’s instructions provided with the corresponding assay kits (DP8018, Jiangsu Zecheng Biotechnology Co., Ltd., Wuxi, China). A total of 12 serum biochemical parameters were measured, including alanine aminotransferase (ALT), aspartate aminotransferase (AST), alkaline phosphatase (ALP), total bilirubin (T-Bil), total protein (TP), albumin (ALB), creatinine (CREA), urea (UREA), glucose (Glu), triglycerides (TG), total cholesterol (TC) and calcium (Ca). Serum biochemical analyses were performed using an automated biochemical analyzer (DP8018 Guangzhou Dongtang Electronic Technology Co., Ltd., Guangzhou, China).

### 2.10. Loading of Ciprofloxacin into Extracellular Vesicles

The antibiotic loading procedure was performed according to the method described by Wu et al. with slight modifications [27]. Briefly, ciprofloxacin (CIP) standard solutions were prepared by serial dilution of a CIP stock solution to generate a range of concentrations. The absorbance of each standard solution was measured at 335 nm using a full wavelength microplate reader, and a standard calibration curve correlating CIP concentration with absorbance was subsequently established.

EVs were mixed with an equal volume of 4 mg/mL CIP solution and incubated at room temperature for 1 h. The mixtures were then subjected to ultrasound treatment in a 35 Hz ultrasonic water bath under ice water cooling conditions, consisting of 30 s sonication followed by 60 s intervals, for a total sonication time of 3 min. Subsequently, the sonicated samples were transferred into 2 mm electroporation cuvettes, with the capacitance set to 50 μF and the resistance set to infinity. Electroporation was performed once at 400 V. The samples were then incubated at room temperature for 2 h to allow recovery of transient membrane pores formed during the loading process. After loading, the samples were transferred into 100 kDa ultrafiltration tubes and centrifuged at 5000 rpm for 10 min to remove free CIP. Sterile PBS was added to the upper chamber of the ultrafiltration tubes, and centrifugation was repeated until no detectable CIP remained in the filtrate. Finally, the purified samples were transferred into 96-well plates, and the absorbance at 335 nm was measured using a full-wavelength microplate reader to calculate the concentration of CIP encapsulated within EVs.

### 2.11. Intracellular Bacterial Killing Assay

The intracellular bacterial killing assay was performed according to the method described by Yuan et al. with slight modifications [28]. Cells were seeded in 24-well plates and cultured until a confluent monolayer was formed. The spent medium was discarded, and the cells were washed with PBS before the addition of fresh maintenance medium. RAW264.7 cells were used to establish an intracellular *Salmonella enterica* serovar Typhimurium SL1344 infection model, whereas Hep-2 cells were used to establish an intracellular *Streptococcus suis* infection model. The final bacterial concentrations were adjusted to 1 × 10^8^ CFU/mL for *Salmonella enterica* serovar Typhimurium SL1344 and 1 × 10^6^ CFU/mL for *S. suis*, followed by incubation at 37 °C for 4 h in a cell culture incubator. Subsequently, the culture medium was removed, and the wells were washed with PBS to eliminate non-adherent bacteria. Cells were then treated for 2 h with maintenance medium containing penicillin (10^4^ μg/mL), streptomycin (10^4^ μg/mL), and vancomycin (100 μg/mL) to eradicate bacteria adhering to the cell surface. After antibiotic treatment, the medium was discarded, the cells were washed with PBS, and fresh maintenance medium was added. Different antibiotic treatments were then applied to the corresponding wells for 6 h as required by the experimental design. In this experiment, ciprofloxacin was used at 10 × MBC, and the MIC and MBC values for each bacterial strain are provided in Supplementary Table S1. For *Salmonella enterica* serovar Typhimurium SL1344, the CIP group received ciprofloxacin at 25 μg/mL, the EV-CIP group received 57.00 μL/mL of EV-CIP, and the EV + CIP group received ciprofloxacin at 25 μg/mL together with 46.96 μL/mL of purified EVs. For *Streptococcus suis* HA9801, the CIP group received ciprofloxacin at 20 μg/mL, the EV-CIP group received 45.60 μL/mL of EV-CIP, and the EV + CIP group received ciprofloxacin at 20 μg/mL together with 38.67 μL/mL of purified EVs. Following incubation, the medium was removed, and the cells were washed with PBS. Subsequently, 200 μL of 0.25% trypsin-EDTA was added to each well for cell lysis for 5 min. The lysates were serially diluted in 96-well plates, and 10 μL aliquots of each dilution were plated onto TSA agar plates. After incubation at 37 °C for 24 h, colony-forming units (CFUs) were enumerated.

### 2.12. Time–Kill Curve Assay

The time–kill curve assay was conducted based on the method established by Jia et al. with slight modifications [29]. In this study, *Salmonella enterica* serovar Typhimurium SL1344 and *Streptococcus suis* QD were used as fluoroquinolone efflux pump-positive bacterial strains. Agarose gel electrophoresis of PCR amplicons confirmed the presence of the corresponding fluoroquinolone efflux pump-associated genes in both strains (Figure S1). The primer sequences are listed in Supplementary Table S2. Bacterial cultures grown to the stationary phase were diluted 1:10 into fresh medium, followed by the addition of different antibiotic treatments as required by the experimental design. In this experiment, ciprofloxacin was used at 10 × MBC, and the MIC and MBC values for each bacterial strain are provided in Supplementary Table S1. For *Salmonella enterica* serovar Typhimurium SL1344, the CIP group received ciprofloxacin at 25 μg/mL, the EV-CIP group received 57.00 μL/mL of EV-CIP, and the EV + CIP group received ciprofloxacin at 25 μg/mL together with 46.96 μL/mL of purified EVs. For *Streptococcus suis* QD, the CIP group received ciprofloxacin at 50 μg/mL, the EV-CIP group received 114.00 μL/mL of EV-CIP, and the EV + CIP group received ciprofloxacin at 50 μg/mL together with 96.68 μL/mL of purified EVs. At 2 h intervals, 100 μL of bacterial suspension was collected, serially diluted, and plated onto TSA agar plates. After incubation at 37 °C for 24 h, CFUs were determined and counted.

### 2.13. Galleria mellonella Larval Survival Assay

The *Galleria mellonella* larval survival assay was performed according to the method described by Nadya et al. with slight modifications [30]. Larvae were maintained at 37 °C, with 10 larvae included in each experimental group. Prior to infection, the larvae were incubated at 37 °C for 2 days to restore their activity. During the experiment, injections were administered into the hemocoel between the last pair of prolegs. In the PBS control group, larvae were injected with 10 μL sterile PBS. In the SL1344 infection group, larvae were injected with 10 μL bacterial suspension containing 1 × 10^6^ CFU/mL SL1344. In the treatment groups, larvae were injected with 10 μL SL1344 suspension mixed with the corresponding drugs. After injection, the larvae were incubated at 37 °C, and larval mortality was recorded every 12 h.

### 2.14. In Vivo Anti-Infection Assay in Mice

All animal procedures were conducted in accordance with the guidelines of the Administration of Affairs Concerning Experimental Animals of China (2017 revision) and were approved by the Experimental Animal Ethics Committee of Henan University of Science and Technology (Approval No. HAUST-023-M0913011). The murine infection model was established according to our previously reported method with slight modifications [31,32]. *Salmonella enterica* serovar Typhimurium SL1344 cultures grown to the stationary phase (1 × 10^9^ CFU/mL) were diluted to 5 × 10^7^ CFU/mL. Twenty-five female BALB/c mice aged 4–6 weeks were randomly assigned to five groups, with five mice per group. Outcome assessment and data analysis were performed in a blinded manner. One group served as the untreated control and received no intervention. Another group was intraperitoneally injected with 200 μL of the *Salmonella enterica* serovar Typhimurium SL1344 suspension. The remaining three groups received 200 ul of the bacterial suspension containing the corresponding ciprofloxacin-based treatment. The CIP group received ciprofloxacin at a dose of 5 mg/kg. The EV + CIP group received ciprofloxacin at 5 mg/kg together with purified EVs at 8.9 μL/g body weight, whereas the EV-CIP group received EV-CIP at 11.4 μL/g body weight. Three days post-infection, all mice were euthanized by intravenous injection of pentobarbital sodium. The liver, spleen, kidneys, and colon were aseptically collected and weighed. Each organ was homogenized in 1 mL sterile PBS under aseptic conditions, followed by serial dilution. Subsequently, 10 μL aliquots of the diluted homogenates were plated onto LA agar plates. After incubation at 37 °C for 24 h, colony-forming units (CFUs) were enumerated.

For histopathological analysis, liver, kidney, spleen, and colon tissues were fixed in 4% paraformaldehyde (pH 7.0) for 36 h. Tissue samples were then embedded in paraffin, sectioned into 4 μm thick slices, and stained with hematoxylin and eosin (H&E). Histological changes were subsequently examined under an optical microscope.

### 2.15. Statistical Analysis

All experiments were independently repeated at least three times. Statistical analyses were performed using one-way analysis of variance (ANOVA) in GraphPad Prism 9.5. For CFU enumeration, colony counts ranging from 10 to 300 were defined as the valid statistical interval, whereas data outside this range were excluded from the analysis. Statistical significance was indicated as follows: ns, not significant; *, *p* < 0.05; **, *p* < 0.01; and ***, *p* < 0.001.

## 3. Results

### 3.1. Characterization and Cellular Internalization of Streptococcus suis T15 Extracellular Vesicles

**Figure 1:**
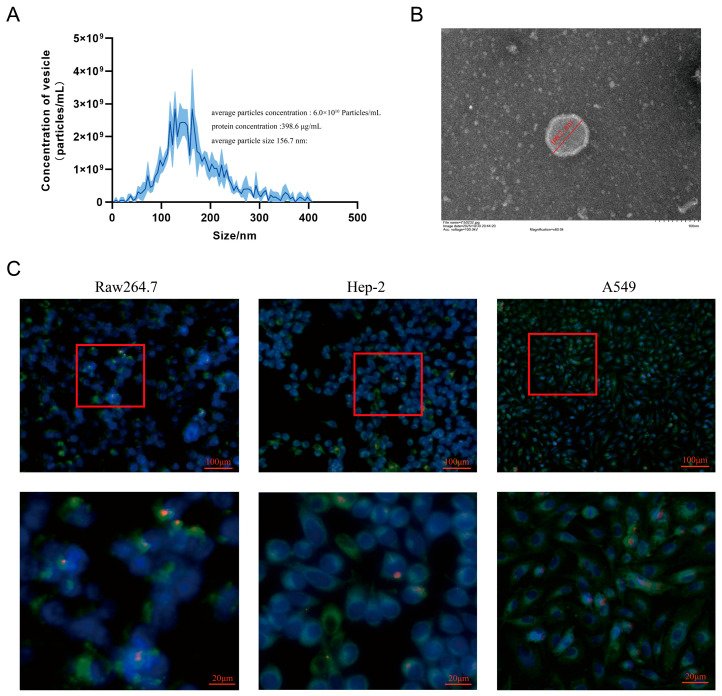
Characterization and cellular internalization of *Streptococcus suis* T15 extracellular vesicles. (**A**) Nanoparticle tracking analysis of EVs. The solid line represents the mean of three independent measurements (*n* = 3), and the shaded area represents the standard error of the mean. (**B**) Transmission electron microscopy observation of EVs. (**C**) Fluorescence microscopy analysis of EV internalization.

Extracellular vesicles (EVs) derived from the avirulent *Streptococcus suis* T15 were isolated and purified using a combination of ultracentrifugation and size exclusion chromatography. As shown in Figure 1A,B, the particle concentration of T15-derived EVs was 6.0 × 10^10^ particles/mL, with an average diameter of 156.7 nm. The total protein concentration of the EVs, determined using a BCA protein assay kit, was 398.6 μg/mL. Subsequently, fluorescently labeled EVs were used to evaluate their cellular internalization capacity in Raw264.7, Hep-2, and A549 cells. As shown in Figure 1C, fluorescence microscopy clearly demonstrated the successful internalization of EVs by all tested cell types.

### 3.2. Safety Evaluation of Streptococcus suis T15-Derived Extracellular Vesicles

**Figure 2:**
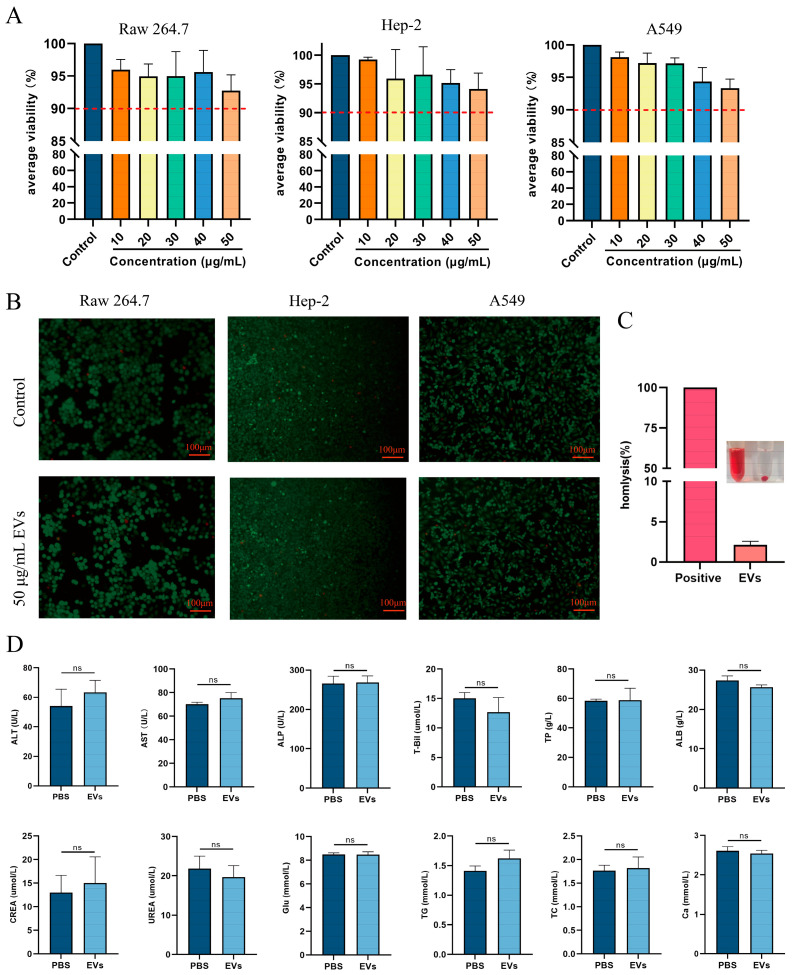
Safety evaluation of *Streptococcus suis* T15-derived extracellular vesicles. (**A**) Cytotoxicity assay; (**B**) live/dead cell double staining analysis; (**C**) hemolysis assay using rabbit erythrocytes; (**D**) serum biochemical analysis in mice following EV administration.

The biosafety of extracellular vesicles (EVs) derived from *Streptococcus suis* T15 was evaluated using Hep-2, Raw264.7, and A549 cells. First, the cytotoxic effects of EVs were assessed using a CCK-8 assay. As shown in Figure 2A, EV concentrations below 50 μg/mL did not induce significant cytotoxicity in any of the tested cell lines. Consistently, fluorescence microscopy analysis revealed similar results (Figure 2B), as preincubation with 50 μg/mL EVs did not result in a significant increase in the number of dead cells. Subsequently, the in vivo biosafety of EVs was further investigated. The rabbit erythrocyte hemolysis assay demonstrated that EVs within this concentration range did not induce hemolytic activity (Figure 2C). In addition, serum biochemical parameters were measured following intraperitoneal injection of EVs into mice. As shown in Figure 2D, all serum biochemical indices remained within the normal range, indicating that EV administration did not cause detectable organ damage and exhibited favorable in vivo biocompatibility.

### 3.3. Loading and Characterization of Ciprofloxacin-Loaded Streptococcus suis Extracellular Vesicles

**Figure 3:**
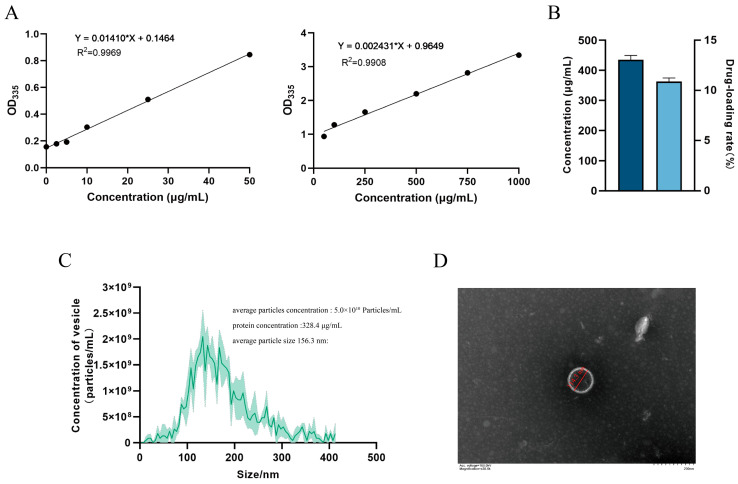
Loading and characterization of ciprofloxacin-loaded *Streptococcus suis* extracellular vesicles. (**A**) Standard calibration curves of ciprofloxacin determined by ultraviolet spectrophotometry; (**B**) drug loading concentration and encapsulation efficiency of EV-CIP; (**C**) particle size distribution of EV-CIP determined by nanoparticle tracking analysis. The solid line represents the mean of three independent measurements (*n* = 3), and the shaded area represents the standard error of the mean. (**D**) Transmission electron microscopy observation of EV-CIP.

As shown in Figure 3A, standard calibration curves for ciprofloxacin (CIP) were successfully established using ultraviolet spectrophotometry. The calibration equations were Y = 0.01410X + 0.1464 (R^2^ = 0.9969) for the concentration range of 2.5–50 μg/mL and Y = 0.002431X + 0.9649 for the concentration range of 50–1000 μg/mL. A combination of ultrasonication and electroporation was employed to load ciprofloxacin into T15-derived EVs. After removal of free ciprofloxacin by ultrafiltration centrifugation, the loading efficiency was evaluated. As shown in Figure 3B, the concentration of ciprofloxacin encapsulated in EVs reached 438.6 μg/mL, with a loading efficiency of 10.96%. The physicochemical properties of ciprofloxacin-loaded EVs (EV-CIP) were further characterized by NTA. As shown in Figure 3C,D, EV-CIP exhibited a particle concentration of 5.0 × 10^10^ particles/mL and an average particle size of 156.3 nm. In addition, BCA protein quantification demonstrated that the total protein concentration of EV-CIP was 328.4 μg/mL.

### 3.4. In Vitro Antibacterial Activity of Vesicle-Delivered Ciprofloxacin (EV-CIP)

**Figure 4:**
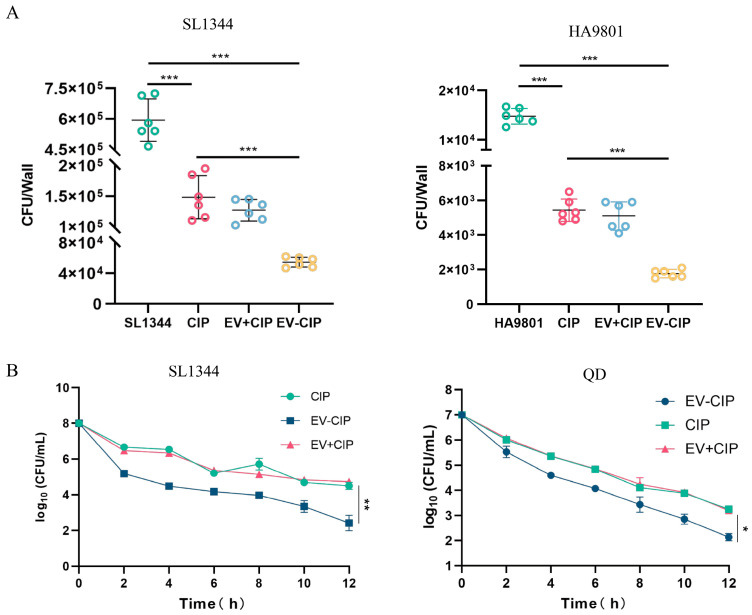
In vitro antibacterial activity of vesicle-delivered Ciprofloxacin (EV-CIP). (**A**) Intracellular bacterial killing assay, (**B**) Antibacterial activity against fluoroquinolone efflux pump-positive strains. Statistical significance was indicated as follows: ns, not significant; *, *p* < 0.05; **, *p* < 0.01; and ***, *p* < 0.001.

The intracellular concentration of antibiotics is often substantially lower than that in the extracellular environment, resulting in reduced bactericidal efficacy against intracellular pathogens. Given the intrinsic cellular internalization capability of EVs, intracellular infection models were established using *Salmonella enterica* serovar Typhimurium SL1344 and *S. suis* HA9801 to evaluate the antibacterial activity of EV-delivered ciprofloxacin (EV-CIP) against intracellular bacteria. As shown in Figure 4A, EV-CIP exhibited significantly enhanced intracellular bacterial clearance compared with free ciprofloxacin (CIP) in both *Salmonella* and *S. suis* infection models. Bacterial efflux pumps can actively expel intracellular antibiotics, thereby reducing the effective intracellular antibiotic concentration and compromising antibacterial activity. Previous studies have suggested that the delivery rate of ciprofloxacin mediated by extracellular vesicles may exceed the drug efflux efficiency mediated by bacterial efflux pumps. Therefore, fluoroquinolone efflux pump-positive strains, including *Salmonella enterica* serovar Typhimurium SL1344 and *Streptococcus suis* QD, were used to evaluate the antibacterial activity of EV-CIP against efflux pump-positive bacteria. Agarose gel electrophoresis of PCR amplicons confirmed that *Salmonella enterica* serovar Typhimurium SL1344 carried the fluoroquinolone efflux pump-associated genes *acrA*, *acrB*, *acrR*, *rob* and *soxS*, whereas *S. suis* QD harbored *SatA* and *SatB* (Figure S1). As shown in Figure 4B, EV-CIP displayed markedly stronger bactericidal activity against efflux pump-positive bacteria than free CIP.

### 3.5. In Vivo Antibacterial Efficacy of Vesicle-Delivered Ciprofloxacin

**Figure 5:**
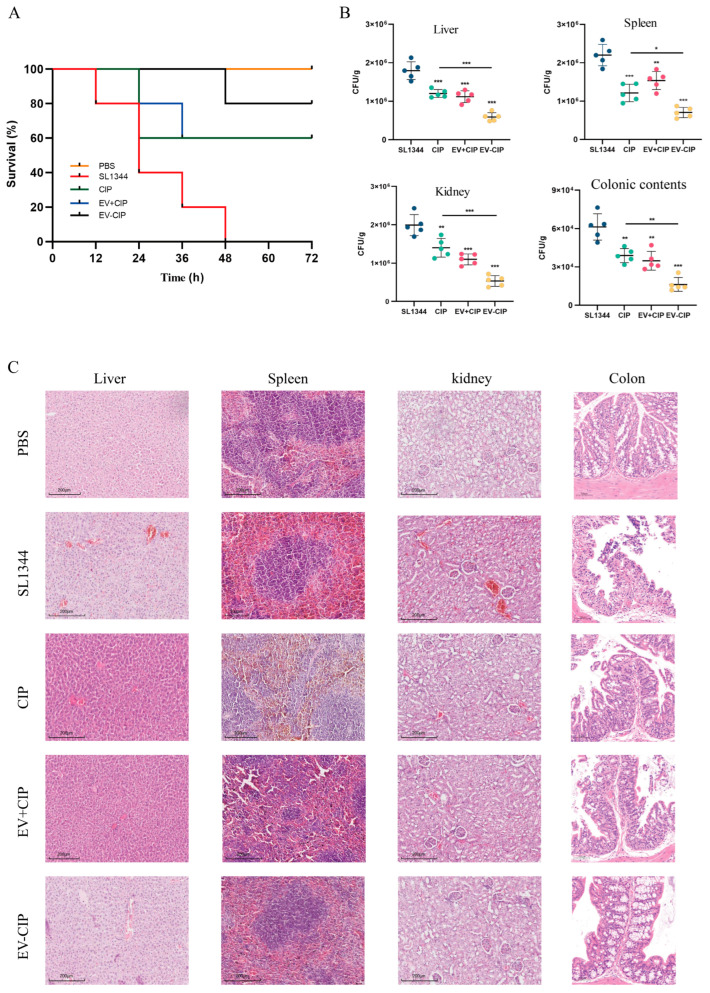
In vivo antibacterial efficacy of vesicle-delivered ciprofloxacin (EV-CIP). (**A**) Survival analysis of *Galleria mellonella* larvae; (**B**) quantification of bacterial loads in mouse organs and colonic contents; (**C**) histopathological analysis of mouse tissues following different treatments. Statistical significance was indicated as follows: ns, not significant; *, *p* < 0.05; **, *p* < 0.01; and ***, *p* < 0.001.

The protective efficacy of EV-CIP was first evaluated using a *Galleria mellonella* infection model. As shown by the survival curves in Figure 5A, all larvae infected with *Salmonella enterica* serovar Typhimurium SL1344 alone died within 48 h. In contrast, treatment with free ciprofloxacin (CIP) or the combination of EVs and CIP (EV + CIP) resulted in a 72 h survival rate of 60%. Notably, treatment with EV-CIP further increased the 72 h survival rate to 80%, demonstrating a significantly improved therapeutic effect compared with free CIP alone. To further assess the in vivo antibacterial activity of EV-CIP, mice were treated for 3 days and subsequently euthanized for organ collection. Bacterial burdens in the liver, spleen, kidneys, and colonic contents were quantified. As shown in Figure 5B, EV-CIP treatment significantly reduced bacterial loads in both organs and colonic contents compared with the free CIP treatment group, indicating enhanced antibacterial efficacy in vivo. Histopathological examination of tissue sections further revealed the therapeutic benefits of EV-CIP (Figure 5C). Following *Salmonella enterica* serovar Typhimurium SL1344 infection, severe pathological damage was observed, including marked hemorrhage around the central veins of the liver, splenic congestion accompanied by blurred boundaries between the red and white pulp and inflammatory cell infiltration, scattered hemorrhagic lesions in the kidneys and extensive damage to the colonic mucosal glands. In contrast, EV-CIP treatment markedly alleviated tissue injury and pathological alterations in infected mice.

## 4. Discussion

Intracellular pathogens reside within host cells and are protected by the cellular membrane barrier, which greatly limits the ability of conventional antibiotics to penetrate cells and effectively eradicate intracellular bacteria. Consequently, intracellular bacterial persistence is widely regarded as one of the major causes of recurrent infections [1]. Although synthetic and natural lipid-based nanocarriers possess several advantages, they still face limitations, including poor stability and susceptibility to degradation. In contrast, bacterial EVs, as naturally derived lipid nanostructures, have emerged as highly promising drug delivery vehicles owing to their intrinsic stability, excellent biocompatibility, and efficient cargo loading capacity. Based on this rationale, the present study successfully employed EVs derived from the avirulent *Streptococcus suis* (*S. suis*) T15 as carriers to construct a ciprofloxacin delivery system, and systematically evaluated its biosafety as well as its therapeutic efficacy both in vitro and in vivo.

In this study, T15-derived EVs were successfully isolated and characterized and were found to be efficiently internalized by multiple host cell types (Figure 1). Similar phenomena have been extensively reported in previous studies. For example, outer membrane vesicles (OMVs) derived from attenuated *Klebsiella pneumoniae* were shown to rapidly deliver doxorubicin into NSCLC A549 cells [33]. These findings suggest that T15 EVs possess intact vesicular structures and effective transmembrane delivery capability. The biosafety of T15-derived EVs was further evaluated using multiple experimental approaches. As shown in Figure 2, EVs exhibited minimal cytotoxicity within the tested concentration range and caused negligible impairment of cellular viability, with no obvious hemolytic activity observed. In addition, serum biochemical analyses in mice demonstrated that T15 EVs did not induce detectable toxicity in major organs, indicating favorable in vivo biocompatibility. These findings are consistent with the results reported by Shi et al., who demonstrated that engineered *Escherichia coli* OMVs exhibited good biosafety profiles in vivo [34]. These results indicate that T15-derived EVs possess intrinsic delivery capabilities. Within the experimental scope of this study, they also exhibited a relatively favorable biosafety profile, providing preliminary support for their potential use as drug-delivery carriers.

A combination of ultrasonication and electroporation was employed to encapsulate ciprofloxacin (CIP) into extracellular vesicles (EVs). Notably, no significant alterations in particle size distribution or total protein concentration were observed before and after drug loading (Figure 3), indicating that the loading procedure did not compromise the structural integrity of the vesicles. The encapsulation efficiency of CIP in T15-derived EVs reached 10.96%. This value differs from previously reported results, in which *Escherichia coli* derived vesicles exhibited a loading efficiency of 41.24% [33], while *Klebsiella pneumoniae* derived vesicles achieved an encapsulation efficiency of 71% Such differences may be attributed to variations in vesicle membrane composition, surface charge characteristics, and other physicochemical properties among vesicles derived from different bacterial species. Taken together, these results demonstrate that T15-derived EVs possess favorable structural stability together with an intrinsic drug loading capacity, supporting their potential as safe and effective antibiotic delivery vehicles.

To further evaluate the therapeutic potential of EV-CIP, intracellular bacterial infection models were established to assess its antibacterial activity against intracellular pathogens. As shown in Figure 4A, EV-CIP exhibited significantly stronger bactericidal activity than either free CIP alone or the combined treatment of EVs and free CIP. Similar findings have been reported by Huang et al. [17], who successfully loaded levofloxacin into Acinetobacter baumannii-derived outer membrane vesicles using a passive loading strategy and demonstrated significantly improved therapeutic efficacy in a murine intestinal infection model. Efflux pumps represent one of the most prevalent mechanisms of bacterial antibiotic resistance, functioning primarily by actively exporting intracellular antibiotics and thereby reducing their effective intracellular concentrations. In the antibacterial assays against fluoroquinolone efflux pump-positive strains (Figure 4), EV-CIP demonstrated markedly greater bactericidal efficacy than free CIP. We speculate that this enhanced activity may be attributed to the vesicle-mediated delivery system, which facilitates increased intracellular accumulation of CIP within bacterial cells, allowing antibiotic concentrations to exceed the drug extrusion capacity of efflux pumps and consequently improve antibacterial efficacy. However, the precise mechanism underlying this effect remains to be elucidated and requires further experimental validation. Antibiotics remain one of the most important tools for controlling bacterial infections in animal production systems. However, the long-term and inappropriate use of antibiotics has accelerated the emergence and dissemination of antimicrobial resistance. The findings of this study indicate that EV-based ciprofloxacin delivery enhances the antibacterial activity of ciprofloxacin against intracellular bacteria and fluoroquinolone-resistant bacteria associated with efflux pump activity. These results provide experimental evidence supporting the feasibility of vesicle-mediated intracellular drug delivery.

The therapeutic outcomes observed in both the *Galleria mellonella* larval infection model and the murine infection model demonstrated that EV-CIP significantly reduced bacterial burdens, alleviated histopathological damage, and improved host survival rates. These findings indicate that EV-CIP maintains favorable stability and therapeutic efficacy in vivo. Moreover, *S. suis* and *Salmonella enterica* serovar Typhimurium are among the most prevalent bacterial pathogens in livestock production systems, causing a wide range of clinical manifestations and substantial economic losses. The EV-based antibiotic delivery system developed in this study enhanced bacterial clearance efficiency, which may contribute to reducing treatment costs associated with bacterial infections. Therefore, these findings support the feasibility of using extracellular vesicles derived from *S. suis* T15 as carriers for intracellular antibiotic delivery. More broadly, this platform may hold promise for antibiotic delivery and the treatment of intracellular bacterial infections in livestock and poultry production.

Despite the promising therapeutic potential demonstrated by the ciprofloxacin delivery system based on EVs derived from the avirulent *S. suis* T15 against intracellular pathogens and fluoroquinolone efflux pump-positive resistant bacteria, several limitations remain in the present study. First, the drug loading efficiency of EV-CIP remained relatively limited. Although the EVs derived from *Streptococcus suis* T15 achieved a ciprofloxacin loading efficiency of 10.96%, the resulting EV-CIP formulation contained ciprofloxacin at a concentration of 438.6 μg/mL, which was sufficient to meet the dosage requirements for both the in vitro cellular assays and the in vivo animal experiments performed in this study. Nevertheless, for the treatment of large economic animals, such as pigs, cattle and sheep, which generally require substantially higher therapeutic doses, the current loading efficiency of EVs remains insufficient. Therefore, further studies are required to optimize EV drug-loading strategies to improve both encapsulation efficiency and delivery performance. Therefore, further studies are required to optimize the drug-loading strategies of EVs in order to improve encapsulation efficiency and delivery performance. Moreover, it should be noted that, although repeated ultrafiltration was performed to remove free ciprofloxacin, the EV-associated drug was not further distinguished as being encapsulated within the vesicle lumen or adsorbed onto the vesicle surface. Accordingly, the measured drug-loading capacity should be interpreted as the total amount of ciprofloxacin associated with EVs rather than the encapsulated fraction alone. Future studies combining surface elution, quantitative analyses using high-performance liquid chromatography (HPLC) and liquid chromatography–tandem mass spectrometry (LC–MS/MS) will help define the spatial distribution of ciprofloxacin within EVs. In addition, the pharmacokinetic characteristics and long-term biosafety of the EV-based delivery system in vivo remain insufficiently understood. Key parameters, including the drug release profile, tissue distribution, circulation stability, targeting efficiency, long-term toxicity, immunogenicity, and potential off-target effects of EV-CIP, have not yet been systematically elucidated. These issues are particularly important for evaluating the translational potential of the platform in target livestock and poultry species. Future studies incorporating in vivo imaging, pharmacokinetic analyses, long-term toxicity assessments, and immunological evaluations will therefore be necessary to further characterize the in vivo behavior, biological fate, and long-term safety of EV-mediated drug delivery systems.

## 5. Conclusions

The results of this study demonstrate that the ciprofloxacin delivery system based on extracellular vesicles derived from the avirulent *Streptococcus suis* T15 exhibited favorable biosafety within the concentration range tested under the present experimental conditions. Compared with free ciprofloxacin, the EV-based delivery system significantly enhanced the antibacterial efficacy of ciprofloxacin against intracellular pathogens and efflux pump-mediated quinolone-resistant bacteria, while also exhibiting superior therapeutic outcomes in animal infection models. These findings provide new experimental evidence supporting EV-mediated intracellular antibiotic delivery and suggest a promising strategy for treating intracellular bacterial infections and enhancing the antibacterial activity of ciprofloxacin against efflux pump-positive strains.

## Data Availability

Data will be made available on request.

## Supplementary Materials

The following supporting information can be downloaded at: https://www.mdpi.com/article/10.3390/ani16142262/s1. Table S1. Minimum inhibitory concentration (MIC) and minimum bactericidal concentration (MBC) of ciprofloxacin against the tested bacterial strains. Table S2. Primer sequences used in this study. Figure S1. Agarose gel electrophoresis analysis of fluoroquinolone efflux pump genes in *Salmonella enterica* serovar Typhimurium SL1344 and *Streptococcus suis* QD. A Agarose gel electrophoresis of fluoroquinolone efflux pump genes in *Salmonella enterica* serovar Typhimurium SL1344. Lane 1, DNA marker Lanes 2–6, *acrA*, *acrR*, *rob*, *soxS*, and *acrB*. B Agarose gel electrophoresis of fluoroquinolone efflux pump genes in *Streptococcus suis* QD. Lane 1, DNA marker; lanes 2 *SatA*, lanes 3 *SatB*.
