# Supplementary material for: In Vitro and In Vivo Antibacterial Efficacy of a Ciprofloxacin Delivery System Based on Streptococcus suis Extracellular Vesicles
Source: Animals (Basel). 2026 Jul 22;16(14):2262. doi: 10.3390/ani16142262 (PMC13405650; doi:10.3390/ani16142262)
Supplement: Supplementary file 1 [file animals-16-02262-s001.zip › animals-4401414-supplementary.pdf]

Supplementary Table S1. Minimum inhibitory concentration (MIC) and minimum bactericidal concentration (MBC) of ciprofloxacin against the tested bacterial strains.

Bacterial strain	MIC (μg/mL)	MBC (μg/mL)
Salmonella enterica serovar Typhimurium SL1344	0.2	2.5
Streptococcus suis HA9801	0.5	2
Streptococcus suis QD	1.25	5

Supplementary Table S2. Primer sequences used in this study.

Primer	Sequence (5′–3′)
SatA-F	CTATTCCTCCCTCCCTTTCTG
SatA-R	GCCATTTTACGTTATAAATGGTACG
SatB-F	CAAACACAAACTGGTTGTGG
SatB-R	CGCTATAAACTGTCCTTTGCTGTG
acrA-F	CCCGCTGACGACTACGC
acrA-R	GCTCTCAGGCAGCTTAGC
acrB-F	GCCAGTACGGTTGCCG
acrB-R	CGCTGACCTTTGAATCCG
acrR-F	CAAGCGCTGGAGACACG
acrR-R	CGATTGCGGAGCAAATAACCAG
rob-F	CGATCGCCGGTTTTG
rob-R	CTGGCATAATTCGCGACC
soxS-F	GCGATCGAACTCGCGG
soxS-R	CCGCTAAACATTGATGTGG

A

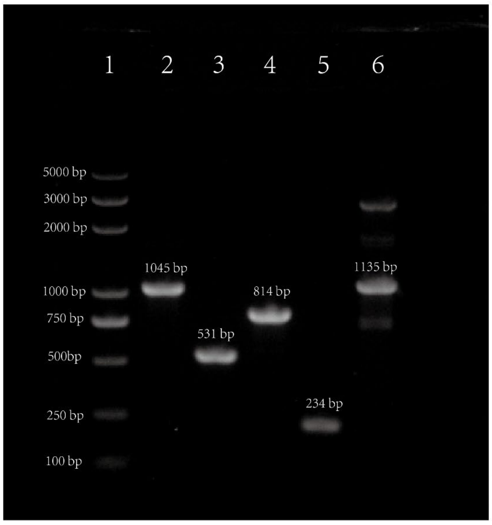

B

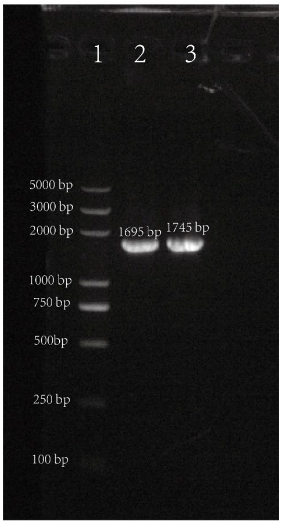

Supplementary Figure S1. Agarose gel electrophoresis analysis of fluoroquinolone efflux pump genes in *Salmonella enterica* serovar Typhimurium SL1344 and *Streptococcus suis* QD. A Agarose gel electrophoresis of fluoroquinolone efflux pump genes in *Salmonella enterica* serovar Typhimurium SL1344. Lane 1, DNA marker Lanes 2–6, acrA, acrR, rob, soxS, and acrB. B Agarose gel electrophoresis of fluoroquinolone efflux pump genes in Streptococcus suis QD. Lane 1, DNA marker; lanes 2 SatA, lanes 3 SatB.
